# Recalcitrant Nummular Eczema in Childhood Responsive to Dupilumab Treatment

**DOI:** 10.7759/cureus.81038

**Published:** 2025-03-23

**Authors:** Luisa R Romiti, Ana B Moraes, Julia-Tatjana Maul, Ricardo Romiti

**Affiliations:** 1 Department of Dermatology, University of Santo Amaro, São Paulo, BRA; 2 Dermatology, Universidade de Caxias do Sul, Caxias do Sul, BRA; 3 Department of Dermatology, University Hospital of Zurich, Zurich, CHE; 4 Department of Dermatology, University of São Paulo, São Paulo, BRA

**Keywords:** atopic dermatitis, childhood, dupilumab, immunobiologics, nummular eczema

## Abstract

Nummular eczema (NE) is a distinctive eczematous dermatitis characterized by an eruption of coin-shaped erythematous, edematous, vesicular, and crusted patches that tend to expand as papulovesicular satellite lesions appear at the periphery and fuse with the main plaque. This pruritic disorder classically affects the extensor aspect of the extremities, trunk, and buttocks. Patients generally report poor quality of life, demanding early and effective treatment. Although topical therapy is the mainstay of management, treatment escalation may be required in severe cases. Dupilumab, a human monoclonal antibody that targets both IL-4 and IL-13, has shown efficacy for moderate to severe atopic dermatitis and other immune-mediated disorders. Here, we report a six-year-old boy with severe and extremely pruritic NE unresponsive to topical and classic systemic treatments, showing complete and maintained remission of all skin signs and symptoms under dupilumab treatment.

## Introduction

Nummular eczema (NE), also named nummular dermatitis or discoid eczema, is an inflammatory chronic and pruriginous disorder characterized by coin-shaped patches typically involving the extremities and, less commonly, the trunk and buttocks [1]. It predominantly affects adults and female patients with a peak incidence of onset in the third and fourth decades of life [2,3]. Lesions may eventually recur or persist for long periods [4]. Topical therapy is the mainstay of management; nevertheless, treatment escalation may be required for severe cases. Dupilumab is a human monoclonal antibody targeting the Th2 immune axis by inhibiting IL-4 and IL-13 and has shown efficacy for moderate to severe atopic dermatitis and sporadic cases of NE in adults [2,5]. We herein present the use of dupilumab in the treatment of severe NE in childhood leading to rapid and sustained resolution of skin inflammation and symptom control.

## Case presentation

**Figure 1:**
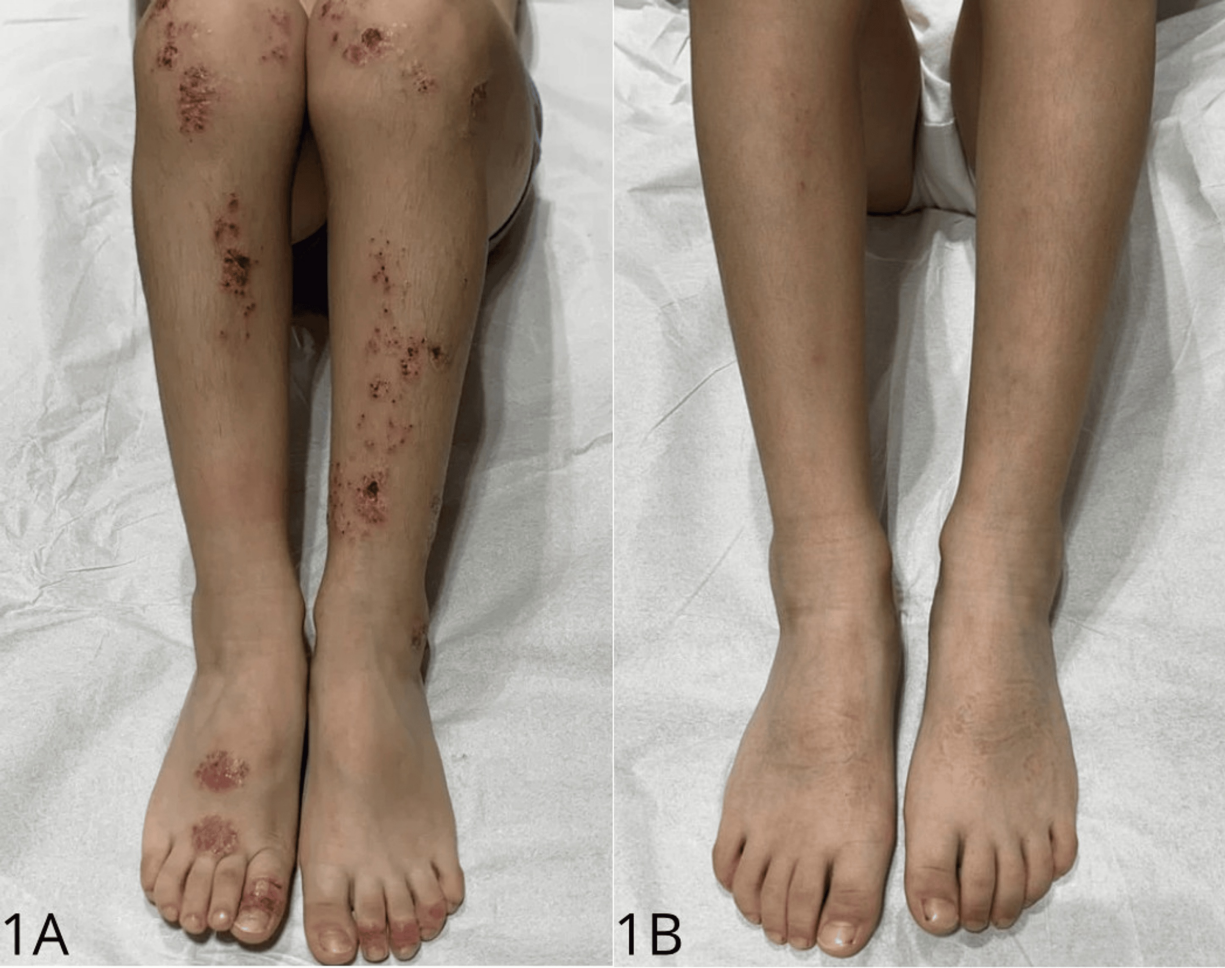
Generalized nummular eczema affecting lower limbs before (1A) and 12 months after dupilumab treatment (1B).

**Figure 2:**
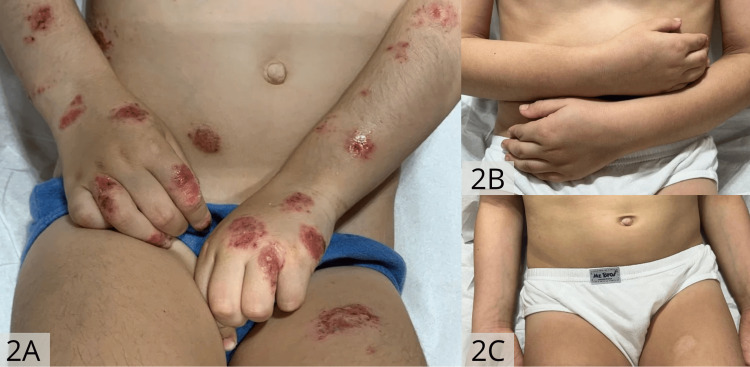
Oozing and bleeding nummular lesions affecting upper limbs and abdomen before (2A) and after dupilumab treatment (2B, 2C).

A six-year-old Brazilian boy presented with a long-standing history of widespread and extremely pruritic eruption since the age of five months. Physical examination revealed numerous nummular, erythematous, and edematous plaques with oozing and crusting affecting the dorsa of the hands and feet, extensor surfaces of the arms and legs, trunk, buttocks, and genital area, severely impairing quality of life (Figures 1A, 2A). No xerosis or other skin signs of atopic dermatitis were observed. No evidence of a family history of atopy was identified. The pruritus Numerical Rating Scale (pNRS) score was nine and atopy patch tests proved negative. Hanifin and Rajka criteria for atopic dermatitis were not present [6].

Previous treatments included long-term topical steroids and calcineurin inhibitors (tacrolimus, pimecrolimus), systemic antibiotics, and a six-month course of oral cyclosporine A (5 mg/Kg/day), with limited improvement. Phototherapy was not available. Due to the recalcitrant lesions and quality of life impairment, subcutaneous injections of dupilumab were initiated at a dose of 300 mg every four weeks. Marked improvement initiated after two weeks of treatment and complete clear skin persisted during a one-year follow-up period with no reported adverse events (Figures 1B, 2B, 2C). The pNRS score decreased to one.

## Discussion

NE is a distinctive eczematous disorder characterized by pruritic and infiltrated coin-shaped or oval pruritic plaques, papules, vesicles, crusts, and desquamation with well-defined borders. Lesions are often exudative and bleeding [2]. A prevalence of two per thousand is estimated worldwide and is more common in the winter and spring months. NE represents an inflammatory skin condition with both type 2 (TH2) and type 3 (TH17) immune signature [7]. Atopic dermatitis frequently has nummular morphology, but in atopy, the lesions tend to be more chronic and lichenified, and specific criteria for diagnosis are classically observed [6]. A higher incidence of atopy is not observed in patients with NE [1,7]. 

The presence of chronic recalcitrant pruritus and substantial quality of life impairments demands early and effective treatment. Topical corticosteroids, alone or in combination with topical antibiotics, are the first-line treatment for NE [1]. In cases involving numerous and recalcitrant lesions, systemic immunosuppressants such as corticosteroids, methotrexate, and cyclosporine are commonly used [2,5]. More recently, a small series of cases of biologics have been reported as effective in adult patients with NE [5]. Dupilumab is a human immunoglobulin subclass 4 (IgG4) monoclonal antibody that targets Th2 immune response and is used in both adults and children with atopic dermatitis, asthma, chronic rhinosinusitis with nasal polyposis, prurigo nodularis, and eosinophilic esophagitis, showing high efficacy and excellent safety profiles [5,7]. Choi et al. reported on six adult cases of NE treated with dupilumab [7]. In total, five patients had a durable response throughout a follow-up period of up to two years and one patient discontinued treatment due to fluctuating response and the development of conjunctivitis. In a multicenter study, evaluating 30 adult patients affected with nummular-like AD treated with dupilumab, significant improvement was observed in all cases after a 16-week period. Conjunctivitis in one patient was the only side effect [8].

In a retrospective chart study, 14 pediatric cases of atopic dermatitis presenting the nummular phenotype were treated with dupilumab. Twelve children demonstrated significant improvement and one case of paradoxical psoriasiform eruption was the only reported side effect [9].

## Conclusions

Here, we report a case of a six-year-old boy with severe and recalcitrant NE unresponsive to topical and conventional systemic treatments. No xerosis or other cutaneous signs of atopic dermatitis could be elicited. The patient achieved marked and sustained remission under dupilumab treatment, and no adverse drug reactions were observed after 12 months of follow-up. The limitation of the study includes the small sample size. Upcoming studies exploring the use of dupilumab in NE will contribute to drawing more precise conclusions. IL-4 and IL-13 blockade might represent a safe and effective treatment modality for children with recalcitrant NE.
